# Role of the Intermediate Filament Protein Peripherin in Health and Disease

**DOI:** 10.3390/ijms232315416

**Published:** 2022-12-06

**Authors:** Roberta Romano, Victoria Stefania Del Fiore, Cecilia Bucci

**Affiliations:** Department of Biological and Environmental Sciences and Technologies, University of Salento, Via Provinciale Lecce-Monteroni n.165, 73100 Lecce, Italy

**Keywords:** intermediate filaments, peripherin, peripheral nervous system, neurons, neurodegenerative diseases, Charcot–Marie–Tooth disease, amyotrophic lateral sclerosis, diabetes, neurodegeneration

## Abstract

Intermediate filaments are the most heterogeneous class among cytoskeletal elements. While some of them have been well-characterized, little is known about peripherin. Peripherin is a class III intermediate filament protein with a specific expression in the peripheral nervous system. Epigenetic modifications are involved in this cell-type-specific expression. Peripherin has important roles in neurite outgrowth and stability, axonal transport, and axonal myelination. Moreover, peripherin interacts with proteins involved in vesicular trafficking, signal transduction, DNA/RNA processing, protein folding, and mitochondrial metabolism, suggesting a role in all these processes. This review collects information regarding peripherin gene regulation, post-translational modifications, and functions and its involvement in the onset of a number of diseases.

## 1. Introduction

The cytoskeleton is an extremely dynamic structure involved in several cellular processes, and it is composed of three types of filaments: microtubules, microfilaments, and intermediate filaments (IFs) [1]. The latter is the most heterogeneous class of filaments, as it comprises plenty of proteins encoded by at least 70 different genes in the human genome. Moreover, the number of IF proteins is further increased by splice variants [2].

Despite this heterogeneity, all the members of this family have a similar structure represented by two non-α-helical domains separated by a central α-helical rod domain. According to in silico structural prediction, in the central region, amino acids are organized in heptad repeats, which form four sub-helices (coil 1A, coil 1B, coil 2A, and coil 2B) that are separated by three linker domains (L1, L12, and L2). However, according to crystallographic studies, the regions indicated as coil 2A, coil L2, and coil 2B seem to constitute a single coiled-coil domain [3,4,5,6,7].

Based on sequence homology and structure, IFs are grouped into six classes: acid and basic keratins (class I and class II); class III, which is the most heterogeneous, comprising vimentin, peripherin, desmin, and glial fibrillary acidic protein (GFAP); class IV, including α-internexin, nestin, synemin, syncoilin, and the neurofilament (NF) proteins (NF-L (light), NF-M (medium), and NF-H (heavy)); class V, comprising lamins; class VI, including the beaded filament structural proteins, Bfsp1 (also called filensin), and Bfsp2 (also called phakinin or CP49) [4,8]. The proteins of the first four classes and class VI are cytosolic, while the lamins of class V are nuclear [1].

IFs derive from the polymerization of two monomers that intertwine in a “coiled-coil” dimer [9]. Then, two dimers associate in an antiparallel manner, forming a tetramer, and eight tetramers form a cylindrical unit filament [10]. Multiple-unit filaments associate with other-unit filaments, giving rise to intermediate filaments [11]. All these processes take place without the involvement of co-factors [12].

While some IFs, such as vimentin, have been well-characterized, little is known about peripherin. This protein shares more than 70% sequence homology with other type III IF proteins such as vimentin, GFAP, and desmin, but only peripherin is specifically expressed in the neurons of the peripheral nervous system [3,13]. Alterations of its structure and assembly are associated with neurodegenerative diseases, but its functions are still poorly understood [2].

Here, we provide an overview of the current knowledge about peripherin and in particular its expression, transcriptional and post-translational regulations, functions, and involvement in some disorders.

## 2. Peripherin Gene

**Figure 1:**
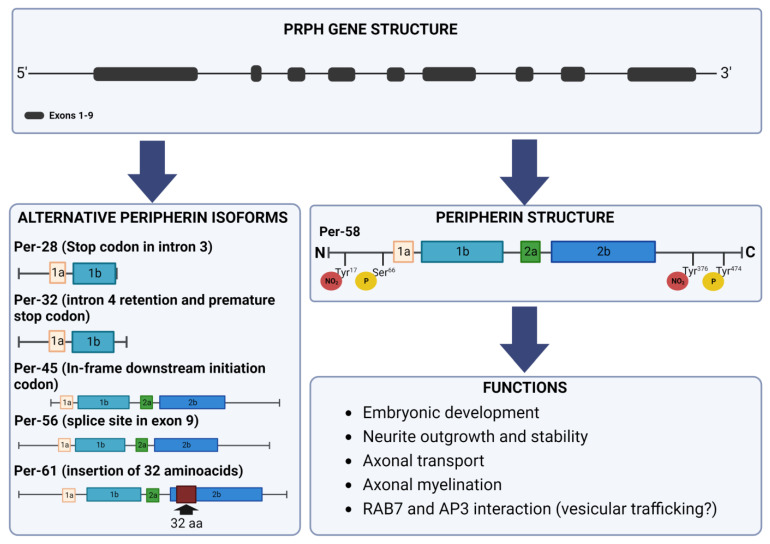
Peripherin gene and protein structure. The peripherin gene is composed of nine exons separated by eight introns. Several isoforms of the peripherin protein exist. Peripherin isoforms are generated by premature stop codon (Per–28), intron retention, premature stop codon (Per–32), in-frame downstream initiation codon (Per–45), alternative splicing (Per–56), and insertion (Per–61). Per–61 is expressed only in mice, Per–32 only in humans. The predominant form of peripherin is Per–58. Peripherin shows several post-translational modifications such as phosphorylation at Ser66 and Tyr474 and nitration at Tyr17 and Tyr376. The known important functions of peripherin in neurons are listed.

The classification of peripherin as a type III intermediate filament protein is based on the complete coding sequence and on the intron–exon pattern of the gene [13,14]. In fact, the structure of the peripherin gene is similar to that of the intermediate filament genes belonging to the same class, while it differs from the neurofilament genes [13,14]. The peripherin gene is composed of nine exons separated by eight introns, and it is conserved between humans, mice, and rats. The nucleotide sequence homology between these species is around 90%, with a difference in the amino acid sequence of only 18 amino acid residues between the human and the rat proteins (Figure 1) [15].

Conserved segments are present in introns 1 and 2 and in the 5′ flanking region [15]. In the 5′ flanking region, the sequence GCTCCTT is identical between humans and rats, and the G was identified as the cap site. For this reason, the G was indicated as base number 1, and the first ATG is located at base +59, in a favorable Kozak context [13,15].

Two other sequences show a high identity between humans, mice, and rats: the first is comprised between −670 and −480 nucleotides; the second, closest to the promoter, is located between −200 and +30 nucleotide [15].

Moreover, the position of introns was compared between humans, mice, and rats, revealing not only the same placement in the three genes but also some differences in the intron length. Indeed, the first intron of the human gene was 100 bp larger, while the eighth intron was 500 bp shorter, compared to those of rodent genes [15].

The comparison between rodent and human genes also highlighted some conserved transcriptional motifs. The NGFNRE (nerve growth factor negative regulatory element) sequence at −172 in humans was similar to that of mice and rats. Moreover, in the proximal 5′ flanking region, two regulatory elements were identified: PER1 and PER3 are added to the previously discovered PER2, a positive cis-element [15,16]. In this region was also identified the B sequence that is common among all type III IF genes [14]. Upstream from the PER elements, there are three protein kinase C and A responsive motifs, AP-2, which shares 90% homology in human and rodent genes [15,17]. Finally, the 5′ flanking region hosts two other conserved sequences: the Hox A5 consensus sequence and one potential binding site for the heat shock transcription factor [15].

These data revealed that the 5′ flanking region of the peripherin gene hosts several evolutionarily conserved domains, including PER1, PER2, PER3, and NGFNRE, AP-2 sites, a homeobox recognition sequence, and a potential binding site for the heat shock transcription factor, all possibly involved in the regulation of peripherin expression.

## 3. Peripherin Expression

Among type III IF proteins, peripherin is the only one selectively expressed in neurons. Its discovery dates back to 1984 as a 57 kDa Triton X-100-insoluble protein observed in mouse neuroblastoma cell lines and the rat pheochromocytoma PC12 cell line, and, in these cells, ^35^S-methionine incorporation into this protein was enhanced following nerve growth factor (NGF) treatment indicating a regulation from this neurotrophin [18]. The name peripherin derives from the results of biochemical studies on different cell lines, primary cultures, and tissues, demonstrating that this protein is mostly expressed in neurons of the peripheral nervous system (PNS) [19].

Its localization in peripheral neurons was further confirmed by other studies by immunofluorescence or in situ hybridization experiments, which demonstrated that peripherin is also expressed in motoneurons [20,21]. Another work evaluated the expression of peripherin during rat development, comparing it to NF-L expression and looking at the central nervous system (CNS) or the PNS. NF-L expression starts at the 25-somite stage, at about 11 days of development, in the ventral horn of the spinal medulla and the posterior part of the rhombencephalon, while peripherin appears a bit later at the 34-somite stage, initially colocalizing with NF-L. After spreading in the rostral and caudal directions, NF-L becomes expressed in the central and peripheral nervous systems, while only the motoneurons of the ventral horn of the spinal medulla, the autonomic ganglionic and preganglionic neurons, and the sensory neurons are positive for peripherin [22]. Moreover, this study demonstrated that peripherin and NF-L start to be expressed during development when migrating neural crest cells reach their destination and terminally differentiate into neurons and that peripherin is expressed in several types of neurons with different functions (motoneurons, sensory neurons, and autonomic neurons), which have in common the fact that their axons reach, at least partially, the region outside the axis constituted by the encephalon and the spinal medulla; these data suggest that peripherin might be important in the recognition of the axonal pathway [22].

During development, peripherin expression is abundant, and several growth factors or cytokines play a role in its regulation. These factors include NGF [23,24], leukemia inhibitory factor (LIF) [25], interleukin-6 [26], and fibroblast growth factor (FGF) [27].

After birth, peripherin expression declines compared to its abundance during development. However, its levels remain high and comparable to those of neurofilaments. Peripherin and NFs are present in a fixed stoichiometry of 4:2:1:1 (NF-L:NF-M:peripherin:NF-H). Moreover, peripherin levels in sciatic axons are drastically reduced following NF-L depletion, and the neurofilament network is altered in the presence of peripherin mutations in transfected SW13vim(-) cells, indicating that peripherin and neurofilaments are functionally interdependent and that peripherin can be considered as a subunit of neurofilaments in the adult PNS [28].

However, a peculiar expression pattern of peripherin and NF-L is present in dorsal root ganglia (DRG). Indeed, immunofluorescence experiments show that, in adult mouse DRGs, there are cells positive for the expression of both NF-L and peripherin as well as cells that do not express NF-L or peripherin. In particular, most of the cells with larger axons express NF-L alone, while cells with smaller axons can be NF-L-negative and peripherin-positive [29]. More recent work confirms that small neurons show a higher expression of peripherin together with synemin M, while NF-L and synemin L characterize larger neurons. Interestingly, in larger neurons, peripherin and synemin M are upregulated after axotomy, suggesting their important role in axonal regeneration. This increase is less strong when vimentin is knocked out, demonstrating the importance of vimentin for the remodeling of IFs after injury [30]. In addition to neurofilaments, peripherin also interacts with syncoilin, another intermediate filament protein. The isoform syncoilin 2 is expressed in the spinal cord and sciatic nerve and modulates the formation of the peripherin filaments network, being necessary for large-caliber motor neurons [31]. These data suggest a different expression pattern and different functions of peripherin in the sensory and motor neurons.

Cell-type-specific expression of peripherin and activation in response to nerve injury depends on both the 5′ flanking region and the intragenic regions. The first 98 bp upstream of the transcription start site includes three regulatory elements, called PER1, PER2, and PER3. PER1 is contained in the TATA box and is held by the DNA-binding proteins prevailing in peripherin-expressing cells. However, the presence of PER1 alone is not sufficient to induce peripherin expression, since peripherin expression levels are also regulated by PER2 and PER3, which function as activator sequences, while PER1 is the principal mediator of neuronal specificity [16]. PER3 is a stronger activator than PER2; it is bound by the transcription factor Sp1, and it stimulates transcription when PER1 is present. Therefore, the regulation of peripherin gene expression is due mostly to the interactions between Sp1 and the proteins binding to PER1 [32]. Some of the regulatory factors present in the 5′ flanking region have been identified: a negative regulatory element (NRE), the deletion of which causes elevated peripherin expression, and two positive regulatory regions fundamental for the expression of peripherin in the PC12 cell line treated with NGF [33].

Regarding the intragenic regions, in cells expressing endogenous peripherin, its expression is lost when intron 1 of the gene is deleted, suggesting the presence of elements fundamental for the full expression of the gene in this region. This sequence is not required for activation of the gene after injury [34].

In order to better understand the mechanism underlying peripherin cell-specific expression, epigenetic modifications were taken into account. In particular, the distribution of DNase I hypersensitive sites (HSS) was analyzed in cells expressing or not expressing peripherin [35]. HSS were found in the active but not in the inactive genes, as the former are characterized by an “open” chromatin conformation, which allows access of cis-acting DNA sequences to trans-acting factors [36]. In peripherin-positive cell lines, at least nine HSS were found (named HSS A–J). Among these, the largest site is represented by HSS G, which is included in the TATA box [35]. In this region, RNA polymerase II can start RNA synthesis at a specific starting point, since DNA is not incorporated into a nucleosomal structure, thanks to the intervention of transcription factors [37]. The majority of HSS are located in the PER elements and in intron 1, confirming that these sequences are involved in the regulation of peripherin gene transcription. The number of HSS near the transcription starting site also reflects the complexity of the regulation of this developmental gene. In peripherin-negative cells, only two or three HSS were identified [35]. Interestingly, in mouse insulinoma βTC cells, eight HSS have been found, but they showed lower intensities by Southern blot analysis compared to peripherin-positive cells. It must be considered that precursors of pancreatic islets express peripherin, so the chromatin structure of βTC cells can represent a preliminary step in peripherin gene activation [35,38]. Interestingly, HSS B is the only region that all peripherin-negative cells have in common; therefore, this region should have a negative effect on the expression of the peripherin gene [35].

Altogether, these data indicate that the open configuration of the gene in peripherin-expressing cells is obtained at a certain stage of the differentiation of sensory, motor, or sympathetic neurons, in response to signals not yet identified, which promotes the interaction with transcription factor, while, in peripherin-negative cells, the gene remains repressed. This mechanism demonstrates a tight regulation of the peripherin gene.

## 4. Peripherin Isoforms

In mice, four isoforms of peripherin were generated by differential splicing: the dominant 58 kDa form Per 58 and three other isoforms named, based on their molecular weight, Per 45, Per 56, and Per 61 (Figure 1) [39,40]. An in-frame downstream initiation codon gives rise to Per 45, a cryptic splice site in exon 9 determines Per 56, and 32 supplementary amino acids in the rod region caused by the retention of intron 4 underlie the formation of Per 61. The latter isoform is not expressed in humans because a truncated frameshifted protein of 32 kDa (Per 32) results from intron 4 retention. However, a human peripherin expressed sequence tag (EST) sequence retaining a part of intron 4 has been identified, indicating that read-through into this intron could occur. In humans, an even shorter isoform exists, named Per 28, which derives from intron 3 and intron 4 retention that originates a stop codon in intron 3 (Figure 1) [41,42].

By comparing the assembly properties of Per 58, Per 56, and Per 61 alone or co-expressed with neurofilaments proteins in SW13 (vim-) cells, it was demonstrated that a normal filamentous network is formed when Per 56 and Per 58 are expressed alone or with neurofilament proteins. On the contrary, the expression of Per 61 does not produce a normal network either, when it is expressed alone or co-expressed with neurofilament proteins. Moreover, Per 61 overexpression in motor neurons determines the formation of aggregates affecting neuronal viability. Per 56 is then capable of establishing a normal network, but the lack of the C-terminal tyrosine phosphorylation could have functional consequences that have not yet been investigated [41].

Per 45 is constitutively expressed in humans and mice, and it is required for the organization of a normal network. Indeed, Per 45 and Per 58 co-assemble in the filamentous network, and the absence of Per 45 causes the formation of irregular filamentous bundles and non-elongated squiggles, despite the ability of Per 58 to self-assemble. Moreover, the correct phenotype is restored in cells that express Per 58 following the expression of Per 45, demonstrating the importance of this isoform in the organization of the appropriate filamentous network [40].

Even though the expression of Per 28 leads to the formation of inclusions in transfected SW13 (vim-) cells and motor neurons, it was associated with mild toxicity, differently from what was demonstrated for Per 61. Importantly, this short isoform is not able to form a filament network [42].

## 5. Post-Translational Modifications

The first evidence about the post-translational modification of peripherin dates back to 1989, when it was demonstrated that peripherin exists as a mixture of phosphorylated and non-phosphorylated forms and that, similarly to vimentin and desmin, phosphorylation sites are located in the amino-terminal half of the protein [43]. In the same year, another study demonstrated that peripherin phosphorylation increases after NGF treatment, suggesting the involvement of this protein in the formation and maintenance of neurites. On the contrary, EGF (Epidermal Growth Factor) or insulin do not influence peripherin phosphorylation, while the effect mediated by NGF is independent of protein kinase A and C activity, suggesting the existence of other involved kinases [44]. Among these, the serine/threonine kinase Akt (also known as protein kinase B) interacts with the head domain of peripherin and phosphorylates peripherin at Ser^66^ (Figure 1) [45].

In addition to serine phosphorylation, peripherin is also phosphorylated at tyrosine^474^, in the carboxy-terminal region of the protein (Figure 1). This further modification is not dependent on NGF treatment and the phospho-dead mutant retains the ability to form a filamentous network, indicating that this modification is not necessary for assembly [46]. The biological role of tyrosine phosphorylation is still unknown. However, peripherin solubility and dynamics are affected by phosphorylation, which is fundamental for intermediate filament network reorganization [47].

Another post-translational modification is nitration, which affects Tyr^17^ and Tyr^376^ (Figure 1). Nitrotyrosination was found not only in the PC12 cell line but also in the rat brain in vivo. This modification increased during NGF-induced differentiation, and the nitrated protein remained closely associated with microtubules, suggesting the role of nitration in stabilizing the cytoskeleton during neuronal differentiation [48,49].

Peripherin is acetylated at Lys^288^ and Lys^398^ and methylated at Arg^72^ and Arg^98^, even though, in this case, the biological functions of these modifications have not yet been investigated [50].

Interestingly, peripherin has been detected as 1 of 13 novel tumor suppressor candidate genes silenced by DNA methylation in hepatocellular carcinoma (HCC). Indeed, it is a target of polycomb repressive complex 2 (PRC2), which has methyltransferase activity [51]. PRC2 interacts with histone deacetylase inhibitors (HDACIs), which is related to tumor suppressor loss [52,53]. Recently, CKD-5 a novel pan-HDACI has been tested for the treatment of HCC by enhancing the effect of sorafenib. This molecule increased peripherin expression in HCC cells, while peripherin silencing after CKD-5 treatment decreased CKD-5-induced apoptosis. The combination therapy with CKD-5 and sorafenib decreased HCC cell viability. CKD-5 probably acts by repressing PRC2 [54].

## 6. Peripherin Functions

Pieces of evidence support the role of peripherin in neurite growth and stability. First, peripherin expression increased concurrently with the initiation of axons during development [22,55,56,57] and in neurons after injury [58,59,60,61]. Second, peripherin silencing in PC12 cells inhibits the initiation, extension, and maintenance of neurites, suggesting the role of peripherin in the architecture of neurons [62]. However, knockout mice develop normally; therefore, peripherin seems to be dispensable for the growth of long myelinating neurons. In these animals, the number of unmyelinated sensory axons in the L5 dorsal roots is reduced, but, in motor neurons, the expression of α-internexin is increased, suggesting that peripherin could be important for the development of a subset of sensory neurons, while in motor neurons α-internexin could compensate for peripherin loss [63].

The importance of peripherin in sensory fiber development is further demonstrated by a recently published study. Peripherin is selectively expressed in the type II spiral ganglion neurons (SGNs) that innervate the electromotile outer hair cells (OHCs) in the post-natal mouse cochlea [64,65]. Peripherin is expressed in both type I and type II SGNs during development, but its expression becomes restricted to type II SGNs shortly after birth [66]. In peripherin knock-out mice, the type II SGNs were disrupted and the OHCs lost their innervation, causing a greater vulnerability to acoustic overstimulation [67]. The reduced synaptic transmission of the type II SGNs, after unilateral cochlear ablation, resulted in decreased peripherin mRNA in the inferior colliculus [68]. In addition to being important for neurite outgrowth, peripherin seems to also be involved in vesicular traffic. Indeed, peripherin interacts with RAB7A, a small GTPase localized to endosomes and involved in the transport to late endosomes and lysosomes and in the biogenesis of lysosomes, phagolysosomes, and autolysosomes [69,70,71,72]. It has been demonstrated that RAB7A is important for peripherin organization and assembly, as modulation of RAB7A expression, by silencing or overexpression, alters the soluble/insoluble ratio of peripherin [73].

There is another peripherin interactor important for late endocytic traffic: the AP-3 adaptor complex, which is involved in the sorting of proteins to the endo-lysosomal system. This protein also interacts with vimentin, and it was demonstrated that the subcellular distribution of AP-3 and lysosomes and the levels of lysosomal proteins LAMP-1 and LAMP-2 were altered when vimentin was silenced. Considering that AP-3 is also a peripherin interactor and that there is a similarity existing between vimentin and peripherin, these data suggest that peripherin might be important for the regulation of late endocytic traffic [74].

Fast axonal transport is another cellular process in which peripherin is involved. Indeed, the transport of endocytic organelles such as lysosomes is altered following peripherin overexpression and NF-L silencing. Lysosomal movement is faster in both directions when NF-L is not expressed, while peripherin overexpression determines a faster lysosomal transport, although only in the anterograde direction [75].

Moreover, in a yeast two-hybrid screen of a mouse brain cDNA library using Per-61 as bait, it was found that peripherin interacts with SNAP25 interacting protein 30 (SIP30), a neuronal protein involved in SNAP receptor-dependent exocytosis [76]. The latter is able to influence peripherin assembly [76]. This interaction suggests a novel role of peripherin in vesicular trafficking. In the same work, it was demonstrated using a yeast two-hybrid screen that peripherin has other interactors involved in vesicular trafficking, signal transduction, DNA/RNA processing, protein folding, and mitochondrial metabolism [76]. Indeed, according to this study, peripherin interacts not only with SIP30 but also with Snapin and Cplx2, which are both involved in vesicular trafficking and synaptic vesicle exocytosis [77,78]. Another interactor identified in this study is HGS (hepatocyte growth factor-regulated tyrosine kinase substrate) [76], which is involved in the intracellular signal transduction mediated by cytokines and growth factors [79]. In addition, peripherin interacts with several zinc finger proteins and HDAC2 [76], which are both involved in transcriptional regulation [80,81], and with UBR3 (E3 ubiquitin protein ligase) [76], which is involved in protein ubiquitination [82]. Finally, several mitochondrial proteins have been identified as peripherin interactors [76]: mitochondrial ribosomal proteins, or mitofusin 1, which mediate mitochondrial fusion [83]. Considering the important functions held by all the proteins identified as peripherin interactors, it was hypothesized that peripherin could participate in all the processes in which these factors are involved [76]. However, all these interactions must be validated, so further studies are necessary to discover their functional meaning to clearly delineate the role of peripherin in these processes (Figure 1) [76].

## 7. Peripherin as a Marker of Different Neuronal Populations

As described before, peripherin is selectively expressed in type II SGNs [66]. Therefore, this protein is widely used as a marker of these nerve fibers [84].

Moreover, peripherin is expressed in the ileum, since it is a marker of enteric neurons [85]. For its expression in this type of neuron, peripherin has also been proposed as a marker for Hirschsprung disease (HD), which is due to the congenital absence of ganglion cells in the distal bowel. Peripherin seems to be superior compared to calretinin and MAP-2 in ruling out HD in small biopsies [86]. Another study recommended the staining of calretinin and peripherin together in patients diagnosed with aganglionosis [87].

Since this protein is also a marker of DRGs, it has been used to investigate the size and distribution of this neuronal population in non-human primates and to compare this information with that obtained in rodent DRG neurons [88].

Another study aimed to investigate the capability of mesenchymal stem cells (MSCs) to differentiate into neuron-like cells in order to repair erectile dysfunction. In this case, peripherin is used to evaluate neuronal differentiation after transplantation [89]. Other examples are represented by human bone marrow-derived MSCs (BMMSCs) and human dental pulp stem cells differentiated in auditory neurons after treatment with microRNA (miRNA)-183 or miRNA-124, respectively. After differentiation, these cells express peripherin [90,91,92]. Increasing the migratory ability of MSCs is fundamental for developing successful cell transplantation therapy, and this can be obtained only by elucidating the mechanisms regulating MSC migration at the molecular level. Remarkably, peripherin silencing in MSCs isolated from bone marrow reduced the ability of these cells to migrate [93].

Remaining in the field of stem cells, Winbo and co-workers were able to establish a functional co-culture of induced pluripotent stem cell (iPSC)-derived sympathetic neurons and cardiomyocytes. Sympathetic neurons stain positive for peripherin, and this specific staining has been exploited to count sympathetic neurons in a semi-automated model that can be used to reliably and quickly estimate sympathetic nervous system nerve fiber density in target tissues [94,95,96]. Moreover, embryonic stem cells have been differentiated into sympathetic neurons expressing peripherin, which represents a useful model to study neuroblastoma pathogenesis [97]. iPSCs were also used to obtain neural crest cells able to differentiate in peripheral neurons. Positive staining for peripherin was used to verify the efficacy of the differentiation [98]. Similarly, neural crest-derived stem cells from human dental pulp have been differentiated into neural-like cells that express peripherin, and skin-derived precursor cells are able to differentiate in peripheral neurons, as demonstrated by their positivity to peripherin staining [99,100]. Peripherin has also been used as a marker of dentary pulp regeneration. Indeed, the regenerated pulp showed a similar distribution pattern of the peripherin neurofilaments to that of the authentic pulp innervation [101]. Neural stem cells have been obtained also from mouse spleen and can differentiate in neural cells in cell culture that stain positive for peripherin [102].

## 8. Role of Peripherin in Diseases

### 8.1. Amyotrophic Lateral Sclerosis (ALS)

**Figure 2:**
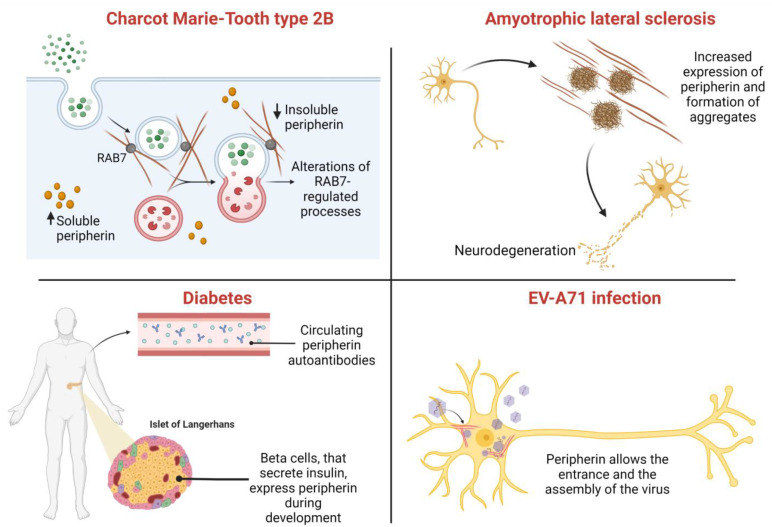
Peripherin is associated with a number of diseases. In Charcot–Marie–Tooth type 2B, disease-causing RAB7A mutations alter peripherin assembly. In amyotrophic lateral sclerosis, the expression of Per-28 is related to the appearance of cytoplasmic aggregates. In diabetic patients, peripherin autoantibodies have been detected is serum. Finally, peripherin promotes the entrance and assembly of EV-A71 enterovirus.

Although the mechanisms of inclusion formation are still poorly understood, peripherin is a major component found in the inclusions of ALS patients (Figure 2) [103,104,105].

Interestingly, heavy tyrosine nitration and phosphorylation of peripherin have been observed in ALS. Nitrated peripherin is only present in the insoluble cytoskeletal fraction; therefore, the increased nitration alters the peripherin soluble/insoluble ratio contributing to the disruption of filament association [106,107].

Moreover, in patients with sporadic forms of ALS, peripherin mutations have been found, which are represented by point mutations and frameshift deletions [108,109,110]. Furthermore, Per 28 isoform is overexpressed and is prone to aggregate [42]. ALS patients are also characterized by an increased expression of peripherin [41]. For this reason, animal models and in vitro models were generated to study the effect of peripherin overexpression on motor neuron degeneration. A late-onset (about 2 years) motor neuron disease with perikaryal and axonal IF inclusions and selective loss of motor neurons was found in a mouse model overexpressing peripherin [111]. Peripherin overexpression is also damaging for cultured cells because it induces apoptotic death [112]. In addition, ALS patients are characterized by the reduction in NF-L mRNA levels in motor neurons; for this reason, double transgenic animal models knockout for NF-L, and overexpressing peripherin are generated. In these animals, motor neuron disease occurs at 6–8 months, with a dramatic loss of motor neurons starting at 5 months [111,113]. Another animal model was generated by overexpressing peripherin and NF-H but knocking down NF-L. Motor neurons of these animals were not damaged and did not show axonal inclusion, probably because of the sequestration of peripherin in the perikaryon [114,115]. These data once again demonstrated the importance of IF protein stoichiometry in neurons.

The appearance of Bunina bodies (BBs) is a key pathological feature of sporadic ALS. Interestingly, these inclusions were found to be immunonegative for Transactivating Response Region (TAR) DNA Binding Protein-43 (TDP-43) but immunopositive for peripherin [116], indicating that peripherin is a marker for these structures.

Moreover, Per 61 is expressed in the motor neurons of transgenic mice expressing the ALS-associated mutation SOD1^G37R^ (Superoxide Dismutase 1) but not in control mice or peripherin-expressing mice. Furthermore, this isoform has been detected in the motor neurons of the lumbar spinal cord from two familial ALS cases, indicating that aberrant splicing can take place in ALS [41].

Another protein mutated in ALS is TDP-43, and it was demonstrated that peripherin regulates the axonal transport of this protein. Therefore, alterations of the peripherin detected in ALS may influence TDP-43 transport in motor neurons [117,118]. Interestingly, a recent study shows that in the spinal cord of ALS patients there are reduced levels of a microRNA (miRNA) named miR-105, and this miRNA is a central regulator of NF-L and peripherin mRNA stability. ALS motor neurons are also characterized by NF-L downregulation, in which miR-105 could have a role, and, maybe, peripherin overexpression could represent a compensatory mechanism in order to attempt to replace NF-L. Therefore, this miRNA is important to maintain IF stoichiometry, and its loss in ALS leads to intermediate filaments dysregulation [119].

Peripherin could also represent a marker of lower motor neuron degeneration, since its level in the cerebrospinal fluid of patients is high [120]. In addition, peripherin might also be used as a biomarker for the diffuse axonal injury caused by traumatic brain injury. Indeed, a study on rats showed a differential expression of this protein between controls and injured rats in which peripherin was found to be overexpressed [121].

These data make peripherin a biomarker both of neurodegeneration and of neuronal injury, which could have an impact on diagnostic accuracy improvement, disease monitoring, and treatment efficacy measurement [122].

### 8.2. Charcot–Marie–Tooth Type 2B

As discussed before, peripherin interacts with the small GTPase RAB7A. Interestingly, this protein is mutated in a neurodegenerative disease called Charcot–Marie–Tooth type 2B (CMT2B), an ulcero-mutilating peripheral neuropathy. Disease-causing RAB7A mutant proteins interact more strongly with peripherin, changing the soluble/insoluble ratio of this intermediate filament protein. Therefore, considering the importance of peripherin in neurite outgrowth after injury, this altered interaction could be relevant for the onset of CMT2B (Figure 2) [73,123].

Moreover, in CMT2B cells, alterations in the late endocytic pathway and mitochondria have been found recently [124,125]. Considering that among peripherin interactors there are molecules regulating not only membrane traffic but also several mitochondrial proteins [76], these pathological phenotypes could be related to alterations in peripherin assembly.

### 8.3. Other Disorders

Recently, a genome-wide association study of sural nerve conduction amplitude and velocity has been performed on the Icelandic population, leading to the individuation of a loss of function peripherin variant that does not allow for the formation of the normal filamentous structure of peripherin, leading to the appearance of punctate protein inclusions. Homozygotes for this variant have a lower sural NC amplitude compared to non-carriers and are at risk of mild, early-onset, sensory-negative, axonal polyneuropathy [126].

Interestingly, peripherin correlates with type 1 diabetes (Figure 2). Patients with autoimmune neuropathies and endocrinopathies as well as nonobese diabetic mouse models show autoantibodies against peripherin [127,128]. The recognized epitope is in the C-terminal tail of Per-58 and Per-61 but not Per-56, which has a C-terminal sequence that differs from those of the other two isoforms [129,130]. Another study shows that 72% of analyzed diabetic patients have peripherin antibodies in serum. These antibodies are directed to phosphorylated peripherin, which represents a major humoral antigen in type 1 diabetes [131]. B cells are an important component of the immune system, and their dysregulation is associated with autoimmune diseases such as type 1 diabetes, even though this disorder seems to be only related to autoreactive T-cells, which infiltrate pancreatic tissue and destroy beta-cells [132]. The mechanism by which B cells contribute to type 1 diabetes pathogenesis is still unknown. Indeed, where and when these cells present the antigen to T-cells is a question that remains to be solved, but, surely, they infiltrate pancreatic islets and secrete peripherin autoantibodies [129,130]. The presence of peripherin antibodies in patients with type 1 diabetes could explain the neuropathy often associated with this disease. Indeed, a model of peripheral neuritis mediated by peripherin-autoreactive B-lymphocytes was recently established in an NOD (nonobese diabetic) mouse model of type 1 diabetes [133]. Peripheral neuropathy is often associated with the onset of severe pain. Interestingly, in Sprague–Dawley rats treated with streptozotocin in order to create a rodent model of type 1 diabetes, cutaneous innervation showed decreased expression of peripherin, possibly contributing to hyperalgesia [134].

Peripherin seems to also have a role in infectious diseases (Figure 2). Indeed, enterovirus-A71 (EV-A71) infects motor neurons and the neuromuscular junction to reach and invade CNS [135]. A recent study demonstrates that peripherin colocalizes with virions and acts as a pro-viral factor: surface-expressed peripherin promotes virus entrance in motor neuron-like and neuroblastoma cell lines, while intracellular peripherin is involved in viral genome replication that interacts with capsid and non-structural viral components. EV-A71 also interacts with a number of peripherin interactors such as the small GTP-binding protein Rac1, which may represent a promising druggable host target [136]. Peripherin, like all type III intermediate filament proteins, does not possess signals for cell membrane recruitment, and the mechanism by which these proteins are transported to the cell surface is still unknown. A recent study suggested that type III intermediate filaments are incorporated into the cell membrane through conformational changes passing by a filamentous to a multimeric structure, and, in this form, they show high affinity for lipid bilayers [137].

## 9. Conclusions

In this review, we discussed the role of peripherin in the nervous system. This protein is specifically expressed in the neurons of the peripheral nervous system, and its gene is finely regulated in order to guarantee such cell-type-specific expression. Peripherin is important in neurite outgrowth and stability and in axonal transport, myelination, and regeneration. Moreover, peripherin interacts with the proteins involved in vesicular trafficking and mitochondrial metabolism, suggesting important functions in these processes as well. Alterations of its expression or assembly have been found in some neurodegenerative disorders, but peripherin is also involved in diabetes and infectious diseases. Nowadays, little is known about peripherin, and further studies are necessary to deeply understand its role in physiological processes and the onset of diseases.

## Data Availability

Not applicable.

## References

[1] Hohmann T., Dehghani F. (2019). The Cytoskeleton—A Complex Interacting Meshwork. Cells.

[2] Margiotta A., Bucci C. (2016). Role of Intermediate Filaments in Vesicular Traffic. Cells.

[3] Fuchs E., Weber K. (1994). Intermediate filaments: Structure, dynamics, function, and disease. Annu. Rev. Biochem..

[4] Herrmann H., Strelkov S.V., Burkhard P., Aebi U. (2009). Intermediate filaments: Primary determinants of cell architecture and plasticity. J. Clin. Investig..

[5] Parry D.A., Strelkov S.V., Burkhard P., Aebi U., Herrmann H. (2007). Towards a molecular description of intermediate filament structure and assembly. Exp. Cell Res..

[6] Geisler N., Weber K. (1982). The amino acid sequence of chicken muscle desmin provides a common structural model for intermediate filaments proteins. EMBO J..

[7] Quax-Jeuken Y.E., Quax W.J., Bloemendal H. (1983). Primary and secondary structure of hamster vimentin predicted from the nucleotide sequence. Proc. Natl. Acad. Sci. USA.

[8] Kornreich M., Avinery R., Malka-Gibor E., Laser-Azogui A., Beck R. (2015). Order and disorder in intermediate filament proteins. FEBS Lett..

[9] Smith T.A., Strelkov S.V., Burkhard P., Aebi U., Parry D.A. (2002). Sequence comparisons of intermediate filament chains: Evidence of a unique functional/structural role for coiled-coil segment 1A and linker L1. J. Struct. Biol..

[10] Sokolova A.V., Kreplak L., Wedig T., Mucke N., Svergun D.I., Herrmann H., Aebi U., Strelkov S.V. (2006). Monitoring intermediate filament assembly by small-angle x-ray scattering reveals the molecular architecture of assembly intermediates. Proc. Natl. Acad. Sci. USA.

[11] Herrmann H., Haner M., Brettel M., Ku N.O., Aebi U. (1999). Characterization of distinct early assembly units of different intermediate filament proteins. J. Mol. Biol..

[12] Herrmann H., Kreplak L., Aebi U. (2004). Isolation, characterization, and in vitro assembly of intermediate filaments. Methods Cell Biol..

[13] Thompson M.A., Ziff E.B. (1989). Structure of the gene encoding peripherin, an NGF-regulated neuronal-specific type III intermediate filament protein. Neuron.

[14] Karpov V., Landon F., Djabali K., Gros F., Portier M.M. (1992). Structure of the mouse gene encoding peripherin: A neuronal intermediate filament protein. Biol. Cell.

[15] Foley J., Ley C.A., Parysek L.M. (1994). The structure of the human peripherin gene (PRPH) and identification of potential regulatory elements. Genomics.

[16] Desmarais D., Filion M., Lapointe L., Royal A. (1992). Cell-specific transcription of the peripherin gene in neuronal cell lines involves a cis-acting element surrounding the TATA box. EMBO J..

[17] Imagawa M., Chiu R., Karin M. (1987). Transcription factor AP-2 mediates induction by two different signal-transduction pathways: Protein kinase C and cAMP. Cell.

[18] Portier M.M., de Nechaud B., Gros F. (1983). Peripherin, a new member of the intermediate filament protein family. Dev. Neurosci..

[19] Portier M.M., Brachet P., Croizat B., Gros F. (1983). Regulation of peripherin in mouse neuroblastoma and rat PC 12 pheochromocytoma cell lines. Dev. Neurosci..

[20] Parysek L.M., Goldman R.D. (1988). Distribution of a novel 57 kDa intermediate filament (IF) protein in the nervous system. J. Neurosci..

[21] Leonard D.G., Gorham J.D., Cole P., Greene L.A., Ziff E.B. (1988). A nerve growth factor-regulated messenger RNA encodes a new intermediate filament protein. J. Cell Biol..

[22] Escurat M., Djabali K., Gumpel M., Gros F., Portier M.M. (1990). Differential expression of two neuronal intermediate-filament proteins, peripherin and the low-molecular-mass neurofilament protein (NF-L), during the development of the rat. J. Neurosci..

[23] Leonard D.G., Ziff E.B., Greene L.A. (1987). Identification and characterization of mRNAs regulated by nerve growth factor in PC12 cells. Mol. Cell. Biol..

[24] Parysek L.M., Goldman R.D. (1987). Characterization of intermediate filaments in PC12 cells. J. Neurosci..

[25] Lecomte M.J., Basseville M., Landon F., Karpov V., Fauquet M. (1998). Transcriptional activation of the mouse peripherin gene by leukemia inhibitory factor: Involvement of STAT proteins. J. Neurochem..

[26] Sterneck E., Kaplan D.R., Johnson P.F. (1996). Interleukin-6 induces expression of peripherin and cooperates with Trk receptor signaling to promote neuronal differentiation in PC12 cells. J. Neurochem..

[27] Choi D.Y., Toledo-Aral J.J., Lin H.Y., Ischenko I., Medina L., Safo P., Mandel G., Levinson S.R., Halegoua S., Hayman M.J. (2001). Fibroblast growth factor receptor 3 induces gene expression primarily through Ras-independent signal transduction pathways. J. Biol. Chem..

[28] Yuan A., Sasaki T., Kumar A., Peterhoff C.M., Rao M.V., Liem R.K., Julien J.P., Nixon R.A. (2012). Peripherin is a subunit of peripheral nerve neurofilaments: Implications for differential vulnerability of CNS and peripheral nervous system axons. J. Neurosci..

[29] Zhao J., Liem R.K. (2016). alpha-Internexin and Peripherin: Expression, Assembly, Functions, and Roles in Disease. Methods Enzymol..

[30] Izmiryan A., Li Z., Nothias F., Eyer J., Paulin D., Soares S., Xue Z. (2021). Inactivation of vimentin in satellite glial cells affects dorsal root ganglion intermediate filament expression and neuronal axon growth in vitro. Mol. Cell. Neurosci..

[31] Clarke W.T., Edwards B., McCullagh K.J., Kemp M.W., Moorwood C., Sherman D.L., Burgess M., Davies K.E. (2010). Syncoilin modulates peripherin filament networks and is necessary for large-calibre motor neurons. J. Cell Sci..

[32] Ferrari N., Desmarais D., Royal A. (1995). Transcriptional activation of the neuronal peripherin-encoding gene depends on a G + C-rich element that binds Sp1 in vitro and in vivo. Gene.

[33] Chang L., Thompson M.A. (1996). Activity of the distal positive element of the peripherin gene is dependent on proteins binding to an Ets-like recognition site and a novel inverted repeat site. J. Biol. Chem..

[34] Uveges T.E., Shan Y., Kramer B.E., Wight D.C., Parysek L.M. (2002). Intron 1 is required for cell type-specific, but not injury-responsive, peripherin gene expression. J. Neurosci..

[35] Karpov V., Fauquet M., Lecomte M.J., Portier M.M. (1996). Three different states of the chromatin structure of the mouse peripherin gene. J. Neurosci. Res..

[36] Gross D.S., Garrard W.T. (1988). Nuclease hypersensitive sites in chromatin. Annu. Rev. Biochem..

[37] Drapkin R., Merino A., Reinberg D. (1993). Regulation of RNA polymerase II transcription. Curr. Opin. Cell Biol..

[38] Escurat M., Djabali K., Huc C., Landon F., Becourt C., Boitard C., Gros F., Portier M.M. (1991). Origin of the beta cells of the islets of Langerhans is further questioned by the expression of neuronal intermediate filament proteins, peripherin and NF-L, in the rat insulinoma RIN5F cell line. Dev. Neurosci..

[39] Landon F., Lemonnier M., Benarous R., Huc C., Fiszman M., Gros F., Portier M.M. (1989). Multiple mRNAs encode peripherin, a neuronal intermediate filament protein. EMBO J..

[40] McLean J., Xiao S., Miyazaki K., Robertson J. (2008). A novel peripherin isoform generated by alternative translation is required for normal filament network formation. J. Neurochem..

[41] Robertson J., Doroudchi M.M., Nguyen M.D., Durham H.D., Strong M.J., Shaw G., Julien J.P., Mushynski W.E. (2003). A neurotoxic peripherin splice variant in a mouse model of ALS. J. Cell Biol..

[42] Xiao S., Tjostheim S., Sanelli T., McLean J.R., Horne P., Fan Y., Ravits J., Strong M.J., Robertson J. (2008). An aggregate-inducing peripherin isoform generated through intron retention is upregulated in amyotrophic lateral sclerosis and associated with disease pathology. J. Neurosci..

[43] Huc C., Escurat M., Djabali K., Derer M., Landon F., Gros F., Portier M.M. (1989). Phosphorylation of peripherin, an intermediate filament protein, in mouse neuroblastoma NIE 115 cell line and in sympathetic neurons. Biochem. Biophys. Res. Commun..

[44] Aletta J.M., Shelanski M.L., Greene L.A. (1989). Phosphorylation of the peripherin 58-kDa neuronal intermediate filament protein. Regulation by nerve growth factor and other agents. J. Biol. Chem..

[45] Konishi H., Namikawa K., Shikata K., Kobatake Y., Tachibana T., Kiyama H. (2007). Identification of peripherin as a Akt substrate in neuron. J. Biol. Chem..

[46] Angelastro J.M., Ho C.L., Frappier T., Liem R.K., Greene L.A. (1998). Peripherin is tyrosine-phosphorylated at its carboxyl-terminal tyrosine. J. Neurochem..

[47] MacTaggart B., Kashina A. (2021). Posttranslational modifications of the cytoskeleton. Cytoskeleton.

[48] Tedeschi G., Cappelletti G., Nonnis S., Taverna F., Negri A., Ronchi C., Ronchi S. (2007). Tyrosine nitration is a novel post-translational modification occurring on the neural intermediate filament protein peripherin. Neurochem. Res..

[49] Cappelletti G., Maggioni M.G., Ronchi C., Maci R., Tedeschi G. (2006). Protein tyrosine nitration is associated with cold- and drug-resistant microtubules in neuronal-like PC12 cells. Neurosci. Lett..

[50] Petzold A. (2022). The 2022 Lady Estelle Wolfson lectureship on neurofilaments. J. Neurochem..

[51] Revill K., Wang T., Lachenmayer A., Kojima K., Harrington A., Li J., Hoshida Y., Llovet J.M., Powers S. (2013). Genome-wide methylation analysis and epigenetic unmasking identify tumor suppressor genes in hepatocellular carcinoma. Gastroenterology.

[52] Kuzmichev A., Nishioka K., Erdjument-Bromage H., Tempst P., Reinberg D. (2002). Histone methyltransferase activity associated with a human multiprotein complex containing the Enhancer of Zeste protein. Genes Dev..

[53] van der Vlag J., Otte A.P. (1999). Transcriptional repression mediated by the human polycomb-group protein EED involves histone deacetylation. Nat. Genet..

[54] Chang Y., Lee Y.B., Cho E.J., Lee J.H., Yu S.J., Kim Y.J., Yoon J.H. (2020). CKD-5, a novel pan-histone deacetylase inhibitor, synergistically enhances the efficacy of sorafenib for hepatocellular carcinoma. BMC Cancer.

[55] Gorham J.D., Baker H., Kegler D., Ziff E.B. (1990). The expression of the neuronal intermediate filament protein peripherin in the rat embryo. Brain Res. Dev. Brain Res..

[56] Troy C.M., Brown K., Greene L.A., Shelanski M.L. (1990). Ontogeny of the neuronal intermediate filament protein, peripherin, in the mouse embryo. Neuroscience.

[57] Undamatla J., Szaro B.G. (2001). Differential expression and localization of neuronal intermediate filament proteins within newly developing neurites in dissociated cultures of Xenopus laevis embryonic spinal cord. Cell Motil. Cytoskelet..

[58] Beaulieu J.M., Kriz J., Julien J.P. (2002). Induction of peripherin expression in subsets of brain neurons after lesion injury or cerebral ischemia. Brain Res..

[59] Oblinger M.M., Wong J., Parysek L.M. (1989). Axotomy-induced changes in the expression of a type III neuronal intermediate filament gene. J. Neurosci..

[60] Troy C.M., Muma N.A., Greene L.A., Price D.L., Shelanski M.L. (1990). Regulation of peripherin and neurofilament expression in regenerating rat motor neurons. Brain Res..

[61] Wong J., Oblinger M.M. (1990). Differential regulation of peripherin and neurofilament gene expression in regenerating rat DRG neurons. J. Neurosci. Res..

[62] Helfand B.T., Mendez M.G., Pugh J., Delsert C., Goldman R.D. (2003). A role for intermediate filaments in determining and maintaining the shape of nerve cells. Mol. Biol. Cell.

[63] Lariviere R.C., Nguyen M.D., Ribeiro-Da-Silva A., Julien J.P. (2002). Reduced number of unmyelinated sensory axons in peripherin null mice. J. Neurochem..

[64] Huang L.C., Thorne P.R., Housley G.D., Montgomery J.M. (2007). Spatiotemporal definition of neurite outgrowth, refinement and retraction in the developing mouse cochlea. Development.

[65] Huang L.C., Barclay M., Lee K., Peter S., Housley G.D., Thorne P.R., Montgomery J.M. (2012). Synaptic profiles during neurite extension, refinement and retraction in the developing cochlea. Neural Dev..

[66] Elliott K.L., Kersigo J., Lee J.H., Jahan I., Pavlinkova G., Fritzsch B., Yamoah E.N. (2021). Developmental Changes in Peripherin-eGFP Expression in Spiral Ganglion Neurons. Front. Cell. Neurosci..

[67] Cederholm J.M.E., Parley K.E., Perera C.J., von Jonquieres G., Pinyon J.L., Julien J.P., Ryugo D.K., Ryan A.F., Housley G.D. (2022). Noise-induced hearing loss vulnerability in type III intermediate filament peripherin gene knockout mice. Front. Neurol..

[68] Kil H.K., Kim K.W., Lee D.H., Lee S.M., Lee C.H., Kim S.Y. (2021). Changes in the Gene Expression Profiles of the Inferior Colliculus Following Unilateral Cochlear Ablation in Adult Rats. Biochem. Genet..

[69] Bucci C., Frunzio R., Chiariotti L., Brown A.L., Rechler M.M., Bruni C.B. (1988). A new member of the ras gene superfamily identified in a rat liver cell line. Nucleic Acids Res..

[70] Bucci C., Thomsen P., Nicoziani P., McCarthy J., van Deurs B. (2000). Rab7: A key to lysosome biogenesis. Mol. Biol. Cell.

[71] Harrison R.E., Bucci C., Vieira O.V., Schroer T.A., Grinstein S. (2003). Phagosomes fuse with late endosomes and/or lysosomes by extension of membrane protrusions along microtubules: Role of Rab7 and RILP. Mol. Cell. Biol..

[72] Jager S., Bucci C., Tanida I., Ueno T., Kominami E., Saftig P., Eskelinen E.L. (2004). Role for Rab7 in maturation of late autophagic vacuoles. J. Cell Sci..

[73] Cogli L., Progida C., Thomas C.L., Spencer-Dene B., Donno C., Schiavo G., Bucci C. (2013). Charcot-Marie-Tooth type 2B disease-causing RAB7A mutant proteins show altered interaction with the neuronal intermediate filament peripherin. Acta Neuropathol..

[74] Styers M.L., Salazar G., Love R., Peden A.A., Kowalczyk A.P., Faundez V. (2004). The endo-lysosomal sorting machinery interacts with the intermediate filament cytoskeleton. Mol. Biol. Cell.

[75] Perrot R., Julien J.P. (2009). Real-time imaging reveals defects of fast axonal transport induced by disorganization of intermediate filaments. FASEB J..

[76] Gentil B.J., McLean J.R., Xiao S., Zhao B., Durham H.D., Robertson J. (2014). A two-hybrid screen identifies an unconventional role for the intermediate filament peripherin in regulating the subcellular distribution of the SNAP25-interacting protein, SIP30. J. Neurochem..

[77] Hazell A.S., Wang D. (2011). Identification of complexin II in astrocytes: A possible regulator of glutamate release in these cells. Biochem. Biophys. Res. Commun..

[78] Ilardi J.M., Mochida S., Sheng Z.H. (1999). Snapin: A SNARE-associated protein implicated in synaptic transmission. Nat. Neurosci..

[79] Rayala S.K., Hollander P., Balasenthil S., Molli P.R., Bean A.J., Vadlamudi R.K., Wang R.A., Kumar R. (2006). Hepatocyte growth factor-regulated tyrosine kinase substrate (HRS) interacts with PELP1 and activates MAPK. J. Biol. Chem..

[80] Cassandri M., Smirnov A., Novelli F., Pitolli C., Agostini M., Malewicz M., Melino G., Raschella G. (2017). Zinc-finger proteins in health and disease. Cell Death Discov..

[81] Kumar V., Kundu S., Singh A., Singh S. (2022). Understanding the Role of Histone Deacetylase and their Inhibitors in Neurodegenerative Disorders: Current Targets and Future Perspective. Curr. Neuropharmacol..

[82] Jiang Z., Zhao Q., Chen L., Luo Y., Shen L., Cao Z., Wang Q. (2022). UBR3 promotes inflammation and apoptosis via DUSP1/p38 pathway in the nucleus pulposus cells of patients with intervertebral disc degeneration. Hum. Cell.

[83] Sidarala V., Zhu J., Levi-D’Ancona E., Pearson G.L., Reck E.C., Walker E.M., Kaufman B.A., Soleimanpour S.A. (2022). Mitofusin 1 and 2 regulation of mitochondrial DNA content is a critical determinant of glucose homeostasis. Nat. Commun..

[84] Kim W.B., Kang K.W., Sharma K., Yi E. (2020). Distribution of Kv3 Subunits in Cochlear Afferent and Efferent Nerve Fibers Implies Distinct Role in Auditory Processing. Exp. Neurobiol..

[85] Grasset E., Puel A., Charpentier J., Klopp P., Christensen J.E., Lelouvier B., Servant F., Blasco-Baque V., Terce F., Burcelin R. (2022). Gut microbiota dysbiosis of type 2 diabetic mice impairs the intestinal daily rhythms of GLP-1 sensitivity. Acta Diabetol..

[86] Chisholm K.M., Longacre T.A. (2016). Utility of Peripherin Versus MAP-2 and Calretinin in the Evaluation of Hirschsprung Disease. Appl. Immunohistochem. Mol. Morphol..

[87] Galazka P., Szylberg L., Bodnar M., Styczynski J., Marszalek A. (2020). Diagnostic Algorithm in Hirschsprung’s Disease: Focus on Immunohistochemistry Markers. In Vivo.

[88] Kudo M., Wupuer S., Kubota S., Seki K. (2021). Distribution of Large and Small Dorsal Root Ganglion Neurons in Common Marmosets. Front. Syst. Neurosci..

[89] Kim J.H., Yun J.H., Song E.S., Kim S.U., Lee H.J., Song Y.S. (2021). Improvement of damaged cavernosa followed by neuron-like differentiation at injured cavernous nerve after transplantation of stem cells seeded on the PLA nanofiber in rats with cavernous nerve injury. Mol. Biol. Rep..

[90] Farnoosh G., Mahmoudian-Sani M.R. (2020). Effects of Growth Factors and the MicroRNA-183 Family on Differentiation of Human Bone Marrow-Derived Mesenchymal Stem Cells Towards Auditory Neuron-Like Cells. Stem Cells Cloning.

[91] Mehri Ghahfarrokhi A., Jami M.S., Hashemzadeh Chaleshtori M. (2020). Upregulation of Neuroprogenitor and Neural Markers via Enforced miR-124 and Growth Factor Treatment. Int. J. Mol. Cell. Med..

[92] Mehri-Ghahfarrokhi A., Pourteymourfard-Tabrizi Z., Farrokhi E., Chaleshtori M.H., Jami M.S. (2019). Increased levels of miR-124 in human dental pulp stem cells alter the expression of neural markers. J. Otol..

[93] Feng Z., Li W., Xia Y., Yu H., Li H., Li K., Mu Y. (2020). The Peripherin Gene Regulates the Migration of Bone Marrow Mesenchymal Stem Cells in Wuzhishan Mini Pigs. Stem Cells Int..

[94] Winbo A., Ramanan S., Eugster E., Jovinge S., Skinner J.R., Montgomery J.M. (2020). Functional coculture of sympathetic neurons and cardiomyocytes derived from human-induced pluripotent stem cells. Am. J. Physiol. Heart Circ. Physiol..

[95] Bleck D., Erdene-Byambadoo L., Brinks R., Schneider M., Pongratz G. (2019). Semi-automated Model to Accurately Counting Sympathetic Nervous Fibers. Bio-Protocol.

[96] Bleck D., Ma L., Erdene-Bymbadoo L., Brinks R., Schneider M., Tian L., Pongratz G. (2019). Introduction and validation of a new semi-automated method to determine sympathetic fiber density in target tissues. PLoS ONE.

[97] Carr-Wilkinson J., Prathalingam N., Pal D., Moad M., Lee N., Sundaresh A., Forgham H., James P., Herbert M., Lako M. (2018). Differentiation of Human Embryonic Stem Cells to Sympathetic Neurons: A Potential Model for Understanding Neuroblastoma Pathogenesis. Stem Cells Int..

[98] Umehara Y., Toyama S., Tominaga M., Matsuda H., Takahashi N., Kamata Y., Niyonsaba F., Ogawa H., Takamori K. (2020). Robust induction of neural crest cells to derive peripheral sensory neurons from human induced pluripotent stem cells. Sci. Rep..

[99] Solis-Castro O.O., Boissonade F.M., Rivolta M.N. (2020). Establishment and neural differentiation of neural crest-derived stem cells from human dental pulp in serum-free conditions. Stem Cells Transl. Med..

[100] Bataille A., Leschiera R., L’Herondelle K., Pennec J.P., Le Goux N., Mignen O., Sakka M., Plee-Gautier E., Brun C., Oddos T. (2020). In Vitro Differentiation of Human Skin-Derived Cells into Functional Sensory Neurons-Like. Cells.

[101] Mounir M.M.F., Rashed F.M., Bukhary S.M. (2019). Regeneration of Neural Networks in Immature Teeth with Non-Vital Pulp Following a Novel Regenerative Procedure. Int. J. Stem Cells.

[102] Tomita K., Ishikawa H. (2019). Existence of Neural Stem Cells in Mouse Spleen. Sci. World J..

[103] Corbo M., Hays A.P. (1992). Peripherin and neurofilament protein coexist in spinal spheroids of motor neuron disease. J. Neuropathol. Exp. Neurol..

[104] Migheli A., Pezzulo T., Attanasio A., Schiffer D. (1993). Peripherin immunoreactive structures in amyotrophic lateral sclerosis. Lab. Investig..

[105] Tu P.H., Raju P., Robinson K.A., Gurney M.E., Trojanowski J.Q., Lee V.M. (1996). Transgenic mice carrying a human mutant superoxide dismutase transgene develop neuronal cytoskeletal pathology resembling human amyotrophic lateral sclerosis lesions. Proc. Natl. Acad. Sci. USA.

[106] Xiao S., McLean J., Robertson J. (2006). Neuronal intermediate filaments and ALS: A new look at an old question. Biochim. Biophys. Acta.

[107] Viedma-Poyatos A., Pajares M.A., Perez-Sala D. (2020). Type III intermediate filaments as targets and effectors of electrophiles and oxidants. Redox Biol..

[108] Corrado L., Carlomagno Y., Falasco L., Mellone S., Godi M., Cova E., Cereda C., Testa L., Mazzini L., D’Alfonso S. (2011). A novel peripherin gene (PRPH) mutation identified in one sporadic amyotrophic lateral sclerosis patient. Neurobiol. Aging.

[109] Gros-Louis F., Larivière R., Gowing G., Laurent S., Camu W., Bouchard J.P., Meininger V., Rouleau G.A., Julien J.P. (2004). A frameshift deletion in peripherin gene associated with amyotrophic lateral sclerosis. J. Biol. Chem..

[110] Leung C.L., He C.Z., Kaufmann P., Chin S.S., Naini A., Liem R.K., Mitsumoto H., Hays A.P. (2004). A pathogenic peripherin gene mutation in a patient with amyotrophic lateral sclerosis. Brain Pathol..

[111] Beaulieu J.M., Nguyen M.D., Julien J.P. (1999). Late onset of motor neurons in mice overexpressing wild-type peripherin. J. Cell Biol..

[112] Robertson J., Beaulieu J.M., Doroudchi M.M., Durham H.D., Julien J.P., Mushynski W.E. (2001). Apoptotic death of neurons exhibiting peripherin aggregates is mediated by the proinflammatory cytokine tumor necrosis factor-alpha. J. Cell Biol..

[113] Beaulieu J.M., Robertson J., Julien J.P. (1999). Interactions between peripherin and neurofilaments in cultured cells: Disruption of peripherin assembly by the NF-M and NF-H subunits. Biochem. Cell Biol..

[114] Beaulieu J.M., Julien J.P. (2003). Peripherin-mediated death of motor neurons rescued by overexpression of neurofilament NF-H proteins. J. Neurochem..

[115] Beaulieu J.M., Jacomy H., Julien J.P. (2000). Formation of intermediate filament protein aggregates with disparate effects in two transgenic mouse models lacking the neurofilament light subunit. J. Neurosci..

[116] Miki Y., Mori F., Seino Y., Tanji K., Yoshizawa T., Kijima H., Shoji M., Wakabayashi K. (2018). Colocalization of Bunina bodies and TDP-43 inclusions in a case of sporadic amyotrophic lateral sclerosis with Lewy body-like hyaline inclusions. Neuropathology.

[117] Muresan V., Ladescu Muresan Z. (2016). Shared Molecular Mechanisms in Alzheimer’s Disease and Amyotrophic Lateral Sclerosis: Neurofilament-Dependent Transport of sAPP, FUS, TDP-43 and SOD1, with Endoplasmic Reticulum-Like Tubules. Neurodegener. Dis..

[118] Oberstadt M., Classen J., Arendt T., Holzer M. (2018). TDP-43 and Cytoskeletal Proteins in ALS. Mol. Neurobiol..

[119] Hawley Z.C.E., Campos-Melo D., Strong M.J. (2019). MiR-105 and miR-9 regulate the mRNA stability of neuronal intermediate filaments. Implications for the pathogenesis of amyotrophic lateral sclerosis (ALS). Brain Res..

[120] Sabbatini D., Raggi F., Ruggero S., Seguso M., Mandrioli J., Cagnin A., Briani C., Toffanin E., Gizzi M., Fortuna A. (2021). Evaluation of peripherin in biofluids of patients with motor neuron diseases. Ann. Clin. Transl. Neurol..

[121] Liang Y., Tong F., Zhang L., Zhu L., Li W., Huang W., Zhao S., He G., Zhou Y. (2019). iTRAQ-based proteomic analysis discovers potential biomarkers of diffuse axonal injury in rats. Brain Res. Bull..

[122] Yuan A., Nixon R.A. (2021). Neurofilament Proteins as Biomarkers to Monitor Neurological Diseases and the Efficacy of Therapies. Front. Neurosci..

[123] Saveri P., De Luca M., Nisi V., Pisciotta C., Romano R., Piscosquito G., Reilly M.M., Polke J.M., Cavallaro T., Fabrizi G.M. (2020). Charcot-Marie-Tooth Type 2B: A New Phenotype Associated with a Novel RAB7A Mutation and Inhibited EGFR Degradation. Cells.

[124] Romano R., Rivellini C., De Luca M., Tonlorenzi R., Beli R., Manganelli F., Nolano M., Santoro L., Eskelinen E.L., Previtali S.C. (2020). Alteration of the late endocytic pathway in Charcot-Marie-Tooth type 2B disease. Cell. Mol. Life Sci..

[125] Gu Y., Guerra F., Hu M., Pope A., Sung K., Yang W., Jetha S., Shoff T.A., Gunatilake T., Dahlkamp O. (2022). Mitochondria dysfunction in Charcot Marie Tooth 2B Peripheral Sensory Neuropathy. Commun. Biol..

[126] Bjornsdottir G., Ivarsdottir E.V., Bjarnadottir K., Benonisdottir S., Gylfadottir S.S., Arnadottir G.A., Benediktsson R., Halldorsson G.H., Helgadottir A., Jonasdottir A. (2019). A PRPH splice-donor variant associates with reduced sural nerve amplitude and risk of peripheral neuropathy. Nat. Commun..

[127] Boitard C., Villa M.C., Becourt C., Gia H.P., Huc C., Sempe P., Portier M.M., Bach J.F. (1992). Peripherin: An islet antigen that is cross-reactive with nonobese diabetic mouse class II gene products. Proc. Natl. Acad. Sci. USA.

[128] Chamberlain J.L., Pittock S.J., Oprescu A.M., Dege C., Apiwattanakul M., Kryzer T.J., Lennon V.A. (2010). Peripherin-IgG association with neurologic and endocrine autoimmunity. J. Autoimmun..

[129] Garabatos N., Alvarez R., Carrillo J., Carrascal J., Izquierdo C., Chapman H.D., Presa M., Mora C., Serreze D.V., Verdaguer J. (2014). In vivo detection of peripherin-specific autoreactive B cells during type 1 diabetes pathogenesis. J. Immunol..

[130] Puertas M.C., Carrillo J., Pastor X., Ampudia R.M., Planas R., Alba A., Bruno R., Pujol-Borrell R., Estanyol J.M., Vives-Pi M. (2007). Peripherin is a relevant neuroendocrine autoantigen recognized by islet-infiltrating B lymphocytes. J. Immunol..

[131] Doran T.M., Morimoto J., Simanski S., Koesema E.J., Clark L.F., Pels K., Stoops S.L., Pugliese A., Skyler J.S., Kodadek T. (2016). Discovery of Phosphorylated Peripherin as a Major Humoral Autoantigen in Type 1 Diabetes Mellitus. Cell Chem. Biol..

[132] Lehuen A., Diana J., Zaccone P., Cooke A. (2010). Immune cell crosstalk in type 1 diabetes. Nat. Rev. Immunol..

[133] Racine J.J., Chapman H.D., Doty R., Cairns B.M., Hines T.J., Tadenev A.L.D., Anderson L.C., Green T., Dyer M.E., Wotton J.M. (2020). T Cells from NOD-PerIg Mice Target Both Pancreatic and Neuronal Tissue. J. Immunol..

[134] Liu C.H., Lan C.T., Chen L.Y., Liao W.C., Ko M.H., Tseng T.J. (2019). Phosphorylation of extracellular signal-regulated kinase 1/2 in subepidermal nerve fibers mediates hyperalgesia following diabetic peripheral neuropathy. Neurotoxicology.

[135] Wang S.M., Liu C.C., Tseng H.W., Wang J.R., Huang C.C., Chen Y.J., Yang Y.J., Lin S.J., Yeh T.F. (1999). Clinical spectrum of enterovirus 71 infection in children in southern Taiwan, with an emphasis on neurological complications. Clin. Infect. Dis..

[136] Lim Z.Q., Ng Q.Y., Oo Y., Chu J.J.H., Ng S.Y., Sze S.K., Alonso S. (2021). Enterovirus-A71 exploits peripherin and Rac1 to invade the central nervous system. EMBO Rep..

[137] Hwang B., Ise H. (2020). Multimeric conformation of type III intermediate filaments but not the filamentous conformation exhibits high affinity to lipid bilayers. Genes Cells.

